# Reduced quenching and extraction time for mammalian cells using filtration and syringe extraction

**DOI:** 10.1016/j.jbiotec.2014.04.014

**Published:** 2014-07-20

**Authors:** Juan A. Hernández Bort, Vinoth Shanmukam, Martin Pabst, Markus Windwarder, Laura Neumann, Ali Alchalabi, Guido Krebiehl, Gunda Koellensperger, Stephan Hann, Denise Sonntag, Friedrich Altmann, Christine Heel, Nicole Borth

**Affiliations:** a ACIB GmbH, Austrian Centre of Industrial Biotechnology, Vienna, Austria; b Department of Biotechnology, University of Natural Resources and Life Sciences, Vienna, Austria; c Department of Chemistry, University of Natural Resources and Life Sciences, Vienna, Austria; d Biocrates Life Sciences, Innsbruck, Austria; e Sandoz GmbH, Tirol, Austria

**Keywords:** ATP, adenosine-5′-triphosphate, ADP, adenosine-5′-diphosphate, AMP, adenosine-5′-monophosphate, UTP, uridine-5′-triphosphate, UDP, uridine-5′-diphosphate, UMP, uridine-5′-monophosphate, GTP, guanosine-5′-triphosphate, GDP, guanosine-5′-diphosphate, GMP, guanosine-5′-monophosphate, UDP-Gal, uridine-5′-diphosphate galactose, UDP-Glc, uridine-5′-diphosphate glucose, UDP-HexNAc, uridine-5′-diphosphate N-acetylhexosamine, Metabolomics, Quenching, Metabolite extraction, Fast filtration, CHO cells

## Abstract

In order to preserve the *in vivo* metabolite levels of cells, a quenching protocol must be quickly executed to avoid degradation of labile metabolites either chemically or biologically. In the case of mammalian cell cultures cultivated in complex media, a wash step previous to quenching is necessary to avoid contamination of the cell pellet with extracellular metabolites, which could distort the real intracellular concentration of metabolites. This is typically achieved either by one or multiple centrifugation/wash steps which delay the time until quenching (even harsh centrifugation requires several minutes for processing until the cells are quenched) or filtration.

In this article, we describe and evaluate a two-step optimized protocol based on fast filtration by use of a vacuum pump for quenching and subsequent extraction of intracellular metabolites from CHO (Chinese hamster ovary) suspension cells, which uses commercially available components. The method allows transfer of washed cells into liquid nitrogen within 10–15 s of sampling and recovers the entire extraction solution volume. It also has the advantage to remove residual filter filaments in the final sample, thus preventing damage to separation columns during subsequent MS analysis. Relative to other methods currently used in the literature, the resulting energy charge of intracellular adenosine nucleotides was increased to 0.94 compared to 0.90 with cold PBS quenching or 0.82 with cold methanol/AMBIC quenching.

## Highlights

• Fast quenching method enabling transfer of washed cells into liquid nitrogen within 15 s.
• Optimized extraction protocol for full recovery of extract solution from cells on the filter.
• Enables accurate analysis of cellular metabolites and improved maintenance for instance of the energy charge of cells.

## 1 Introduction

In recent years, different sophisticated protocols have been developed for the quantification of intracellular metabolites (Bennett et al., 2008; Buchholz et al., 2001; Dietmair et al., 2010; Neubauer et al., 2012; Pabst et al., 2010; Sellick et al., 2011) due to the increasing interest in more rational metabolic engineering and control tools for the optimization of cell lines and processes (Dietmair et al., 2012a,b; Martínez et al., 2013; Murabito et al., 2009; Schilling et al., 2000; Sheikh et al., 2005). The use of reconstructed genome-scale models requires sufficient and reliable experimental data to predict the cellular needs of high-producer cell lines (Chung et al., 2010; Licona-Cassani et al., 2012; Selvarasu et al., 2012) and to assist with rational prediction of necessary changes in media composition, feeding strategy and process control (D’Huys et al., 2012; Martínez et al., 2013; Selvarasu et al., 2009, 2012; Zamorano et al., 2012).

To ensure that the measured data are as close to the real *in vivo* values as possible, an efficient quenching protocol should: (i) ensure a fast and complete blockage of any intracellular metabolic reaction, as most intermediates have high conversion rates (Canelas et al., 2008; de Koning and van Dam, 1992; Weibel et al., 1974), (ii) avoid contamination of the sample with extracellular metabolites present in the supernatant, (iii) lose none of the intracellular substances through leaky membranes. It has proven very difficult to achieve all of these requirements to perfection, so that most protocols try to keep a reasonable balance of preventing all three reasons of metabolite loss or contamination.

Several authors (Pabst et al., 2010; Sellick et al., 2009) designed quenching protocols in which the cell broth was directly quenched using cold solvent mixtures after sampling, as their lower freezing points allow using them below −20 °C. Even though these methods were successfully implemented for yeast and bacteria (de Jonge et al., 2012; Gonzalez et al., 1997; Moritz et al., 2000) their direct application to mammalian cells provokes some controversies due to higher leakage of intracellular metabolites, as mammalian cells lack a cell wall. Sellick (Sellick et al., 2009) and later Sengupta (Sengupta et al., 2011) used different additives to the cold methanol quenching solvent showing an increase in metabolite recovery. However, these results proved difficult to transfer between labs (Dietmair et al., 2010) and indeed, as expected, resulted in membrane damage and consequently uncontrolled leakage of intracellular metabolites.

A less aggressive quenching method uses direct dilution of the cell broth with ice-cold PBS (Pabst et al., 2010), which prevents leakage while at the same time diluting extracellular contaminants, however the final mixture does not achieve temperatures below 0 °C, which does not ensure quenching of all enzymatic reactions and it cannot be excluded that molecules from the supernatant contaminate the intracellular concentrations analyzed.

To avoid metabolite leakage, fast filtration was proposed as a washing step prior to quenching with liquid N_2_ (Volmer et al., 2011a,b). Using an in-house made filter module, a determined amount of cells were passed through a filter and rapidly washed with 0.9% (w/w) NaCl. The entire procedure took between 30 and 60 s and allows obtaining cells free from any contaminating metabolites present in the cultivation media. Quenching with liquid N_2_ has the advantage to rapidly freeze samples below −100 °C, thus avoiding further metabolic conversion (Buziol et al., 2002; Sellick et al., 2011). However, the system so far required individually made equipment and thus necessitated fine tuning of the individual components to identify optimal conditions of handling and use, making it difficult to transfer to other labs.

Despite the fast quenching step, the subsequent extraction of metabolites from cells captured on a filter is time consuming, as the filters have to be cut to fit into a tube, to be centrifuged together with the extraction solvent, and the final liquid extract is separated from the filter by decantation. The filter retains some fluid which makes it difficult to recover the entire volume added for the extraction. This can be partially resolved by using a suction system coupled to a column (Volmer et al., 2011a,b).

It was reported, that several methods for the intracellular metabolite extraction using solvents like hot methanol (Shryock et al., 1986), cold methanol (de Jonge et al., 2012; Volmer et al., 2011b), boiling ethanol (Gonzalez et al., 1997), chloroform/methanol (de Koning and van Dam, 1992), acid (Ryll and Wagner, 1991), alkali (Nissom et al., 2006) and hot water (Bolten et al., 2007) were successful. Though, to attain the maximum recovery of metabolites, cold methanol (Cao et al., 2011; Shin et al., 2010; Volmer et al., 2011a) and cold acetonitrile (Dietmair et al., 2010) are more preferred in mammalian cells.

All in all, the selection of a tailored protocol will depend on the cell characteristics, the metabolites required for the study or analytical equipment availability. In this work, we developed a modified protocol for fast filtration, washing and quenching using readily available parts. The entire procedure routinely takes less than 15 s to transfer washed cells into liquid nitrogen. For subsequent extraction either immediately after quenching or after storage, different cold solutions were compared and evaluated by LC–ESI-MS and FIA-MS–MS analyses of nucleotides and amino acids. The method is benchmarked against other quenching and extraction methods described in the literature and found to preserve the energy charge of AMP/ADP/ATP at the highest value (0.94 vs 0.90 and 0.82).

## 2 Materials and methods

### 2.1 Cell line and cultivation media

CHO-K1 suspension cell lines adapted to growth in glutamine free media (Bort et al., 2010) were cultivated in 500 ml spinner flasks using CD CHO media (Gibco, Invitrogen, Carlsbad, CA, USA) under 7% CO_2_ and incubated at 37 °C with constant stirring at 50 rpm. Viability and cell counts were measured on a Vi-Cell analyzer (Beckman Coulter Inc., Fullerton, CA) based on the trypan-blue dye exclusion method.

### 2.2 Filtration and quenching

**Fig. 1:**
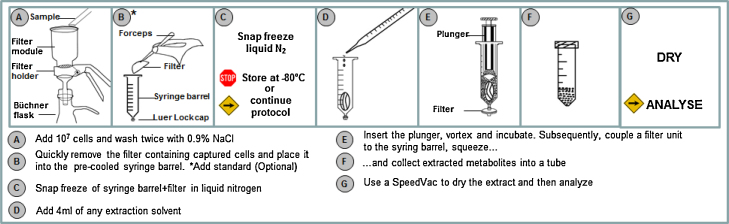
Flow chart of steps for fast quenching and extraction.

For each sample, after humidification of the filter membrane (PALL A/D Glass Fibre, 47 mm, New York, USA) with 10 ml of 0.9% (w/w) NaCl, a total of 10^7^ cells harvested from a suspension culture were filtered using a standard filtration holder (Millipore, MA, USA) and immediately washed with 30 ml 0.9% (w/w) NaCl solution under controlled vacuum at 10 mbar (Controller CVC 3000 with W-B 6C, Vacuumbrand, Germany) to remove extracellular contaminating substances. The filter membrane with the captured cells was transferred into a 20 ml syringe barrel (20 ml BD™ Syringe with Luer-Lock™ connection, Becton Dickinson, NJ, USA) closed with a cap (Fig. 1). At this step a standard solution could be added (see f.i. Neubauer et al., 2012), before freezing of samples in liquid nitrogen, followed either by storage at −80 °C or immediate extraction.

Cold PBS quenching (Pabst et al., 2010) and cold methanol/AMBIC quenching (Sellick et al., 2009) were carried out exactly as published and compared to fast filtration by analysis of nucleotides and sugar nucleotides.

### 2.3 Metabolites extraction

Following quenching in liquid N_2_, 8 ml of 50% (v/v) cold methanol were added into the syringe barrel. The plunger was carefully reinserted into the syringe barrel after removing the Luer-Lock™ cap and slightly pushing on the plunger to remove the excess air inside. The syringe barrel was relocked with a Luer-Lock™ cap and vortexed for 15 s. The cap was removed and a 0.45 μm filter membrane unit (Millex HV-Durapore PVDF filter) was attached to the syringe. The syringe plunger was squeezed to transfer the extraction solution into a fresh tube. The entire extracted volume (8 ml or as specified), was dried with a Thermo Savant SPD121P Speed Vac Concentrator equipment (Thermo Fischer Scientific, MA, USA) and stored at −80 °C for further quantification of metabolites.

### 2.4 LC–ESI-MS analysis for nucleotides

The sample extraction and analysis of the nucleotides and nucleotide sugars were performed as described in Pabst et al. (2010) and Taschwer et al. (2012). The data were interpreted using Bruker's Data Analysis 4.0 software, using integration of selected ion chromatograms. All compounds were referred to the internal 13C10-ATP Standard (Sigma Aldrich, USA) for quantification. To allow correction for different ionization and detection efficiency of the compounds, equimolar mixtures of representative nucleotides were generated and measured under the same conditions (data not shown). Adenosines and Guanosines standards were purchased from Larova, Germany, Uridines and Cytidines from Sigma Aldrich, USA.

Energy charge was calculated as established by Atkinson (1968):

$$\text{Energy charge}=\frac{\text{ATP}+0.5\text{ADP}}{\text{ATP}+\text{ADP}+\text{AMP}}$$

### 2.5 Lactate dehydrogenase analysis

After cell broth filtration with different vacuum pressures, the concentration of lactate dehydrogenase (LDH) in the flow-through filtrate was determined with an LDH Test Kit (Roche Diagnostics, Germany) following manufacturer instructions, in order to quantify cellular membrane damage of filtered CHO cells.

### 2.6 FIA-MS–MS Analysis

Biocrates’ commercially available KIT plates (Innsbruck, Austria) were used for the quantification of amino acids, hexose (glucose), and biogenic amines. The fully automated assay was based on PITC (phenylisothiocyanate)-derivatization in the presence of internal standards followed by FIA-MS/MS (acylcarnitines, lipids, and hexose) and LC/MS (amino acids, biogenic amines) using an AB SCIEX 4000 QTrap™ mass spectrometer (AB SCIEX, Darmstadt, Germany) with electrospray ionization. The experimental metabolomics measurement technique is described in detail in the BIOCRATES AbsoluteIDQTM p180 KIT assay manual.

## 3 Results and discussion

### 3.1 Adaptation of protocol and optimization of vacuum for minimal leakage of cells

**Fig. 2:**
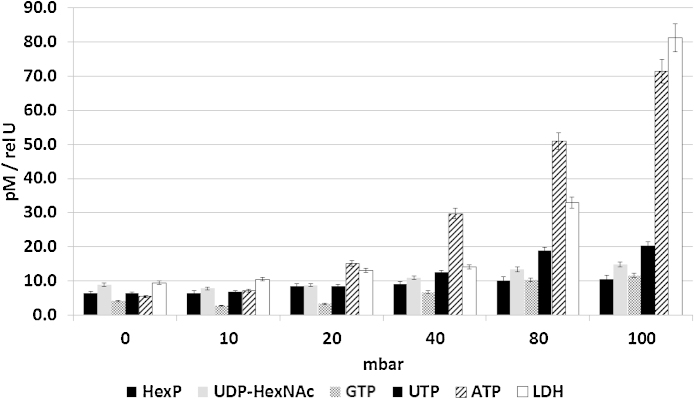
Influence of vacuum on leakage of intracellular molecules into filtrate. Both low molecular weight substances and an intracellular protein (LDH) were analyzed in the filtered wash solution (*n* = 3). Results for LDH are given as relative units. Concentrations of released small molecules start to increase at 20 mbar vacuum pressure, so that 10 mbar was chosen for all subsequent experiments.

Fast filtration has been shown to be an efficient method for the separation of mammalian cells from complex cultivation media before quenching (Volmer et al., 2011a). We adapted the method to use only commercially available components (Fig. 1) to enable its application in any standard lab environment. A vacuum pump is used at a defined vacuum strength sufficient to increase filtration speed, but not so high as to cause damage to the cell membrane, which would result in leakage of intracellular metabolites. Therefore, a broad range of vacuum pressures (500, 80, 40, 20 and 10 mbar below the ambient air pressure) was tested and the flow-through of the wash-step analyzed for presence of lactate dehydrogenase (LDH) (as an example of a large molecule released from cells upon death, analyzed by an enzyme assay) and selected nucleotides and nucleotide sugars using LC–ESI-MS (as examples for small molecular weight metabolites with rapid turnover) (Fig. 2). As a control, three samples were filtered and washed without any vacuum at ambient air pressure (samples labeled 0-mbar). In this case the filtration and washing step took over 50 s, while the procedure took approximately 10 s when using 10 mbar of vacuum. As the filter clogged when 5 × 10^7^ cells were applied (Murabito et al., 2009), which drastically increased the time between filtration and quenching, the applied number of cells used was 10^7^ per filter for all samples. Leakage of small metabolites, especially of ATP, started to increase from 20 mbar, while larger test substances such as LDH started to significantly increase from 40 mbar onwards, similar to values published by Volmer et al. (2011a). The differences can be explained by the use of different equipment parts, however, with the protocol here described all components are readily available commercially, can be connected using standard lab equipment and require no handicraft work.

### 3.2 Completeness of quenching of metabolite conversion

**Fig. 3:**
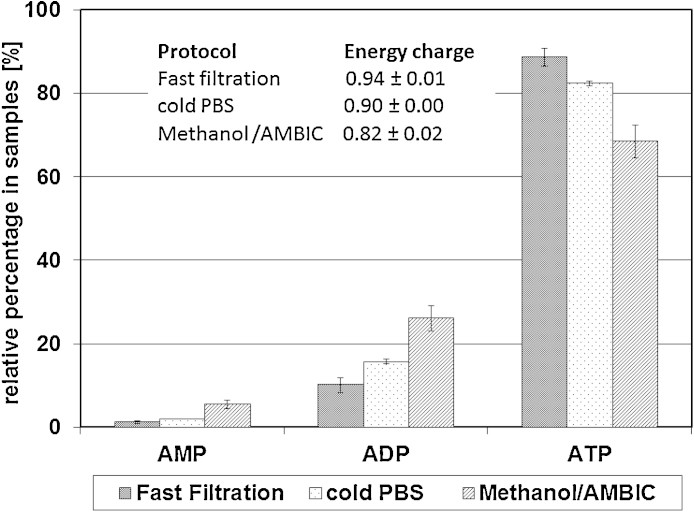
Comparison of efficiency of quenching. After quenching, all samples were extracted and analyzed for nucleotides (*n* = 4). The relative distribution of AMP/ADP/ATP for each protocol is shown as well as the calculated energy charge.

To assess the speed and completeness of blocking all metabolic conversions in the cells, the energy charge (Ec) of cells quenched by fast filtration was compared to that of cells quenched by previously published methods, either with cold phosphate-buffered saline (pH 7.4, 0.5 °C) or with cold 60% methanol containing 0.85% (w/v) ammonium bicarbonate (AMBIC) (pH 7.4; −20 °C). For all approaches cells were isolated from the same shaker flask and each quenching procedure was performed twice by two independent operators (total *n* = 4). Depending on the nutrient state of the cells, the Ec of viable cells is expected to lie between 0.7 and 0.95 (Andersen and von Meyenburg, 1977; Atkinson, 1968; Bontemps et al., 1993; Cook et al., 1977; Sellick et al., 2011). Assuming that all samples coming from the same culture have an identical Ec, differences between quenching methods would be due to incomplete quenching and ongoing consumption of ATP by intracellular enzymes, resulting in reduced ATP and increased ADP or AMP concentrations and thus a reduced Ec (Fig. 3). The maintenance of metabolites with high conversion rates was best in the fast filtration/liquid nitrogen quenching protocol. The absolute recovery of sugar nucleotides (which have a lower conversion rate than nucleotide bi- and triphosphates) by the different methods was on average 100 for the fast filtration protocol, 150 for cold PBS and 20 for the methanol/AMBIC protocol (relative values, data not shown), indicating that with the methanol/AMBIC protocol significant loss of intracellular metabolites occurs due to leakage caused by membrane solubilisation by the methanol. The higher values for the cold PBS protocol are more difficult to explain, as they could be caused either by a low level of leakage occurring in the fast filtration protocol, or by incomplete removal of contaminating molecules derived from dead cells from the culture supernatant in the cold PBS method.

### 3.3 Optimization of metabolite extraction

In addition to the quenching method, the extraction of the metabolites and the subsequent sample treatment play an important role for the determination of metabolite concentrations. To provide an optimal extraction method, different volumes (4, 6, 8 ml) and ratios of the extraction solvent (methanol/ddH_2_O; 100/0, 80/20, 50/50, 0/100) were analyzed by FIA-MS–MS. In general, the extraction protocols consist of disruption of cells by addition of the extraction solvent, dissolution of all cellular molecules and drying of the extract to obtain higher concentrations and better storability. Obviously, the drying requires harsh conditions that might affect the stability and recovery of metabolites. To analyze these effects, samples of different volume (1000, 500 and 250 μl) were dried in a Speed Vac and re-dissolved in 250 μl of the corresponding extraction solvent. These results were compared to samples which were directly frozen without Speed Vac treatment. This also yielded results on the effect of concentrating the metabolite amounts (concentration factor due to re-suspension: 4×, 2×, 1×).

**Fig. 4:**
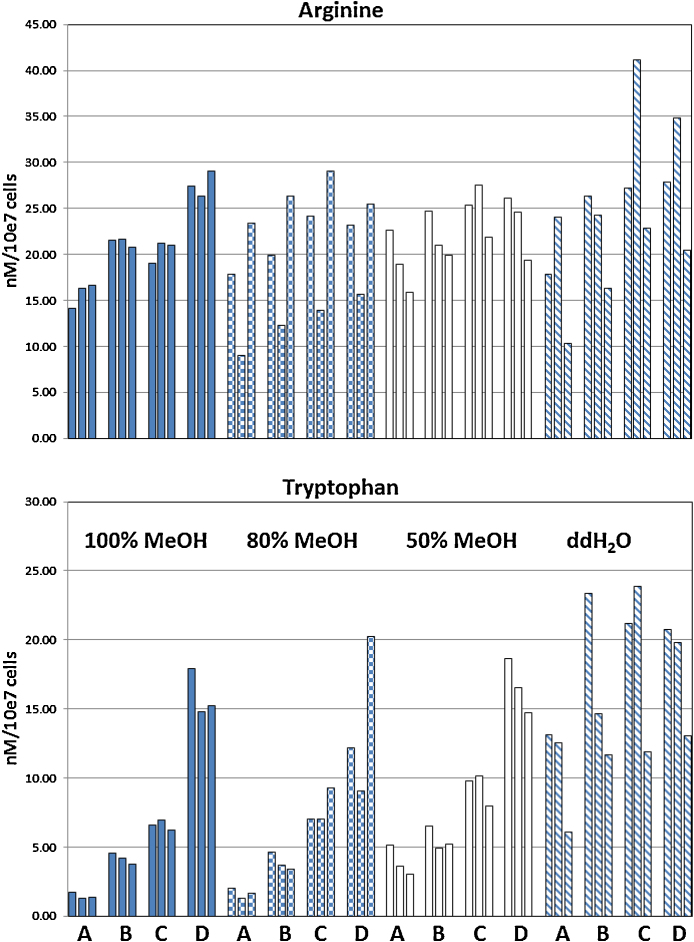
Effects of extraction volume, drying and extraction solvents on intracellular arginine and tryptophan concentration: 10^7^ cells were extracted using 8 ml (left bar within each group), 6 ml (middle bar) or 4 ml (right bar) of extraction solvent as specified in the graph. After extraction with the respective total volume, different amounts of extract were dried and reconstituted in 250 μl of the respective solvent. Volumes dried were 1000 μl (A), 500 μl (B) and 250 μl (C). Finally an aliquot of the extraction solution was directly analyzed without drying (D). Intracellular amino acid amounts are given as nm per 10^7^ cells.

Representative results on amino acid analyses are shown on the example of arginine and tryptophan (Fig. 4). The extraction volume used in the syringe had the least effect on the results, while the volume used for drying had a significant effect on most amino acids, especially on methionine, which was only detectable when analyzed directly and completely disappeared in the dried extracts. The larger the volume subjected to drying, the lower the observed concentration per cell. Aspartate, isoleucine, proline, phenylalanine and lysine were the least sensitive to this effect, showing only insignificant differences between the dried and directly analyzed samples. Arginine showed modest effects, while tryptophan had significant loss during drying (Fig. 4). Results were the most consistent with 100% methanol as extraction solvent, while all other solvents showed higher variability between different handling procedures and also between replicates. This might be due to less efficient cell disruption and solution of metabolites. However, the effect of drying was also the most pronounced with 100% MeOH. Using distilled water for cell disruption worked very well when larger extraction volumes were used (8 ml or 6 ml), however, with 4 ml the recovered concentration of extracts was lower, most likely due to incomplete cell disruption in the lower volume. Detailed results for all amino acids are available as Supplement 1. Additional metabolites analyzed included biogenic amines and hexoses (Suppl. 1). Hexose gave similar results to the amino acids, with the most reliable extraction achieved by 100% methanol, while for the biogenic amines more differential results were observed: putrescine, creatinine and sarcosine behave similar to the amino acids, while acetylornithine, methionine-sulfoxide and to a lesser degree taurine were only well extracted in the methanol containing extraction solutions, with hardly any extraction possible when using water. Finally for spermidine the situation was reversed, with good extraction only possible with water and not at all with any of the methanol containing extractants. These results demonstrate the necessity to use multiple extraction solutions depending on the desired metabolites if a complete analysis of the metabolome of cells is required.

## 4 Conclusion

This work corroborates fast filtration as a tailored approach to be used before quenching and extraction of cells cultivated in complex media. Depending on filtration equipment and cell type, the choice of an appropriate vacuum pressure is necessary in order to reduce leakage of metabolites. Using commercially available plastic syringes which have a smooth inner surface, the filter can be quickly transferred into liquid nitrogen, without the need to cut it, which will lead to loss of time and potentially metabolites. Typical transfer times of washed cells into liquid nitrogen were below 15 s, with approximately 30 s handling time for filter change until the next replicate can be processed.

Also, the recovery of metabolites during extraction is easier and faster using the syringe. Traces or particles of the filter or cells can be removed by coupling a 0.45 μm filter membrane unit, thus increasing the life span of analytical columns. The entire protocol allows the operator to extract metabolites in less than 2 min, thus enabling the handling of a large number of samples for bioprocess analyses.

Regarding further processing, speedvac treatment has shown a strong effect on the stability of several metabolites analyzed compared to samples which were directly frozen after extraction. The reason for this negative effect is likely to be the warm temperatures during the speedvac treatment and the time which is needed for this process. That effect was most prominent in amino acids after extraction with any methanol-solvent. In summary, the best results on the determination of metabolite concentrations in CHO cells were obtained when 4 ml of 100% methanol were used for extraction and aliquots of each sample were directly analyzed. Nevertheless, for other methods, speedvac treatment is required to remove solvents for protection of columns. In this case the use of an internal standard that contains the same metabolites as expected in the sample (Neubauer et al., 2012) is required. In addition, other classes of analytes may require different extraction solutions. Here again the speed of this method would be of advantage, as both the sampling procedure onto the filters as well as the extraction procedure are easy and fast to enable generation of several sample loaded filters and subsequent extraction with multiple extract solutions.

## References

[bib0005] Andersen K.B., von Meyenburg K. (1977). Charges of nicotinamide adenine nucleotides and adenylate energy charge as regulatory parameters of the metabolism in *Escherichia coli*. J. Biol. Chem..

[bib0010] Atkinson D.E. (1968). The energy charge of the adenylate pool as a regulatory parameter. Interaction with feedback modifiers. Biochemistry.

[bib0015] Bennett B.D., Yuan J., Kimball E.H., Rabinowitz J.D. (2008). Absolute quantitation of intracellular metabolite concentrations by an isotope ratio-based approach. Nat. Protoc..

[bib0020] Bolten C.J., Kiefer P., Letisse F., Portais J.C., Wittmann C. (2007). Sampling for metabolome analysis of microorganisms. Anal. Chem..

[bib0025] Bontemps F., Vincent M.F., Van den Berghe G. (1993). Mechanisms of elevation of adenosine levels in anoxic hepatocytes. Biochem. J..

[bib0030] Bort J.A., Stern B., Borth N. (2010). CHO-K1 host cells adapted to growth in glutamine-free medium by FACS-assisted evolution. Biotechnol. J..

[bib0035] Buchholz A., Takors R., Wandrey C. (2001). Quantification of intracellular metabolites in *Escherichia coli* K12 using liquid chromatographic–electrospray ionization tandem mass spectrometric techniques. Anal. Biochem..

[bib0040] Buziol S., Bashir I., Baumeister A., Claassen W., Noisommit-Rizzi N., Mailinger W., Reuss M. (2002). New bioreactor-coupled rapid stopped-flow sampling technique for measurements of metabolite dynamics on a subsecond time scale. Biotechnol. Bioeng..

[bib0045] Canelas A.B., Ras C., ten Perick A., van Dam J.C., Heijnen J.J., van Gulik W.M. (2008). Leakage-free rapid quenching technique for yeast metabolomics. Metabolomics.

[bib0050] Cao B., Aa J., Wang G., Wu X., Liu L., Li M., Shi J., Wang X., Zhao C., Zheng T. (2011). GC–TOFMS analysis of metabolites in adherent MDCK cells and a novel strategy for identifying intracellular metabolic markers for use as cell amount indicators in data normalization. Anal. Bioanal. Chem..

[bib0055] Chung B.K., Selvarasu S., Andrea C., Ryu J., Lee H., Ahn J., Lee D.Y. (2010). Genome-scale metabolic reconstruction and in silico analysis of methylotrophic yeast *Pichia pastoris* for strain improvement. Microb. Cell Fact..

[bib0060] Cook G.A., Sullivan A.C., Ontko J.A. (1977). Influences of intracellular pyridine nucleotide redox states on fatty acid synthesis in isolated rat hepatocytes. Arch. Biochem. Biophys..

[bib0065] D’Huys P.J., Lule I., Vercammen D., Anné J., Van Impe J.F., Bernaerts K. (2012). Genome-scale metabolic flux analysis of *Streptomyces lividans* growing on a complex medium. J. Biotechnol..

[bib0070] de Jonge L.P., Douma R.D., Heijnen J.J., van Gulik W.M. (2012). Optimization of cold methanol quenching for quantitative metabolomics of *Penicillium chrysogenum*. Metabolomics.

[bib0075] de Koning W., van Dam K. (1992). A method for the determination of changes of glycolytic metabolites in yeast on a subsecond time scale using extraction at neutral pH. Anal. Biochem..

[bib0080] Dietmair S., Hodson M.P., Quek L.-E., Timmins N.E., Chrysanthopoulos P., Jacob S.S., Gray P., Nielsen L.K. (2012). Metabolite profiling of CHO cells with different growth characteristics. Biotechnol. Bioeng..

[bib0085] Dietmair S., Timmins N.E., Gray P.P., Nielsen L.K., Krömer J.O. (2010). Towards quantitative metabolomics of mammalian cells: development of a metabolite extraction protocol. Anal. Biochem..

[bib0090] Dietmair S., Nielsen L.K., Timmins N.E. (2012). Mammalian cells as biopharmaceutical production hosts in the age of omics. Biotechnol. J..

[bib0095] Gonzalez B., François J., Renaud M. (1997). A rapid and reliable method for metabolite extraction in yeast using boiling buffered ethanol. Yeast.

[bib0100] Licona-Cassani C., Marcellin E., Quek L.E., Jacob S., Nielsen L.K. (2012). Reconstruction of the *Saccharopolyspora erythraea* genome-scale model and its use for enhancing erythromycin production. Anton. Leeuw..

[bib0105] Martínez V.S., Dietmair S., Quek L.E., Hodson M.P., Gray P., Nielsen L.K. (2013). Flux balance analysis of CHO cells before and after a metabolic switch from lactate production to consumption. Biotechnol. Bioeng..

[bib0110] Moritz B., Striegel K., De Graaf A.A., Sahm H. (2000). Kinetic properties of the glucose-6-phosphate and 6-phosphogluconate dehydrogenases from *Corynebacterium glutamicum* and their application for predicting pentose phosphate pathway flux in vivo. Eur. J. Biochem..

[bib0115] Murabito E., Simeonidis E., Smallbone K., Swinton J. (2009). Capturing the essence of a metabolic network: a flux balance analysis approach. J. Theor. Biol..

[bib0120] Neubauer S., Haberhauer-Troyer C., Klavins K., Russmayer H., Steiger M.G., Gasser B., Sauer M., Mattanovich D., Hann S., Koellensperger G. (2012). U(13) C cell extract of *Pichia pastoris* – a powerful tool for evaluation of sample preparation in metabolomics. J. Sep. Sci..

[bib0125] Nissom P.M., Sanny A., Kok Y.J., Hiang Y.T., Chuah S.H., Shing T.K., Lee Y.Y., Wong K.T., Hu W.S., Sim M.Y. (2006). Transcriptome and proteome profiling to understanding the biology of high productivity CHO cells. Mol. Biotechnol..

[bib0130] Pabst M., Grass J., Fischl R., Léonard R., Jin C., Hinterkörner G., Borth N., Altmann F. (2010). Nucleotide and nucleotide sugar analysis by liquid chromatography–electrospray ionization-mass spectrometry on surface-conditioned porous graphitic carbon. Anal. Chem..

[bib0135] Ryll T., Wagner R. (1991). Improved ion-pair high-performance liquid chromatographic method for the quantification of a wide variety of nucleotides and sugar-nucleotides in animal cells. J. Chromatogr..

[bib0140] Schilling C.H., Edwards J.S., Letscher D., Palsson B. (2000). Combining pathway analysis with flux balance analysis for the comprehensive study of metabolic systems. Biotechnol. Bioeng..

[bib0145] Sellick C.A., Hansen R., Maqsood A.R., Dunn W.B., Stephens G.M., Goodacre R., Dickson A.J. (2009). Effective quenching processes for physiologically valid metabolite profiling of suspension cultured Mammalian cells. Anal. Chem..

[bib0150] Sellick C.A., Hansen R., Stephens G.M., Goodacre R., Dickson A.J. (2011). Metabolite extraction from suspension-cultured mammalian cells for global metabolite profiling. Nat. Protoc..

[bib0155] Selvarasu S., Ho Y.S., Chong W.P., Wong N.S., Yusufi F.N., Lee Y.Y., Yap M.G., Lee D.Y. (2012). Combined in silico modeling and metabolomics analysis to characterize fed-batch CHO cell culture. Biotechnol. Bioeng..

[bib0160] Selvarasu S., Wong V.V., Karimi I.A., Lee D.Y. (2009). Elucidation of metabolism in hybridoma cells grown in fed-batch culture by genome-scale modeling. Biotechnol. Bioeng..

[bib0165] Sengupta N., Rose S.T., Morgan J.A. (2011). Metabolic flux analysis of CHO cell metabolism in the late non-growth phase. Biotechnol. Bioeng..

[bib0170] Sheikh K., Förster J., Nielsen L.K. (2005). Modeling hybridoma cell metabolism using a generic genome-scale metabolic model of *Mus musculus*. Biotechnol. Prog..

[bib0175] Shin M.H., Lee d.Y., Liu K.H., Fiehn O., Kim K.H. (2010). Evaluation of sampling and extraction methodologies for the global metabolic profiling of *Saccharophagus degradans*. Anal. Chem..

[bib0180] Shryock J.C., Rubio R., Berne R.M. (1986). Extraction of adenine nucleotides from cultured endothelial cells. Anal. Biochem..

[bib0185] Taschwer M., Hackl M., Hernández Bort J.A., Leitner C., Kumar N., Puc U., Grass J., Papst M., Kunert R., Altmann F. (2012). Growth, productivity and protein glycosylation in a CHO EpoFc producer cell line adapted to glutamine-free growth. J. Biotechnol..

[bib0190] Volmer M., Gettmann J., Scholz S., Büntemeyer H., Noll T. (2011). A method for metabolomic sampling of suspended animal cells using fast filtration. BMC Proc..

[bib0195] Volmer M., Northoff S., Scholz S., Thüte T., Büntemeyer H., Noll T. (2011). Fast filtration for metabolome sampling of suspended animal cells. Biotechnol. Lett..

[bib0200] Weibel K.E., Mor J.R., Fiechter A. (1974). Rapid sampling of yeast cells and automated assays of adenylate, citrate, pyruvate and glucose-6-phosphate pools. Anal. Biochem..

[bib0205] Zamorano F., Vande Wouwer A., Jungers R.M., Bastin G. (2012). Dynamic metabolic models of CHO cell cultures through minimal sets of elementary flux modes. J. Biotechnol..

